# Supplementary figures and images for: LSD1 defines the fiber type-selective responsiveness to environmental stress in skeletal muscle
Source: eLife. 2023 Jan 25;12:e84618. doi: 10.7554/eLife.84618 (PMC9876571; doi:10.7554/eLife.84618)

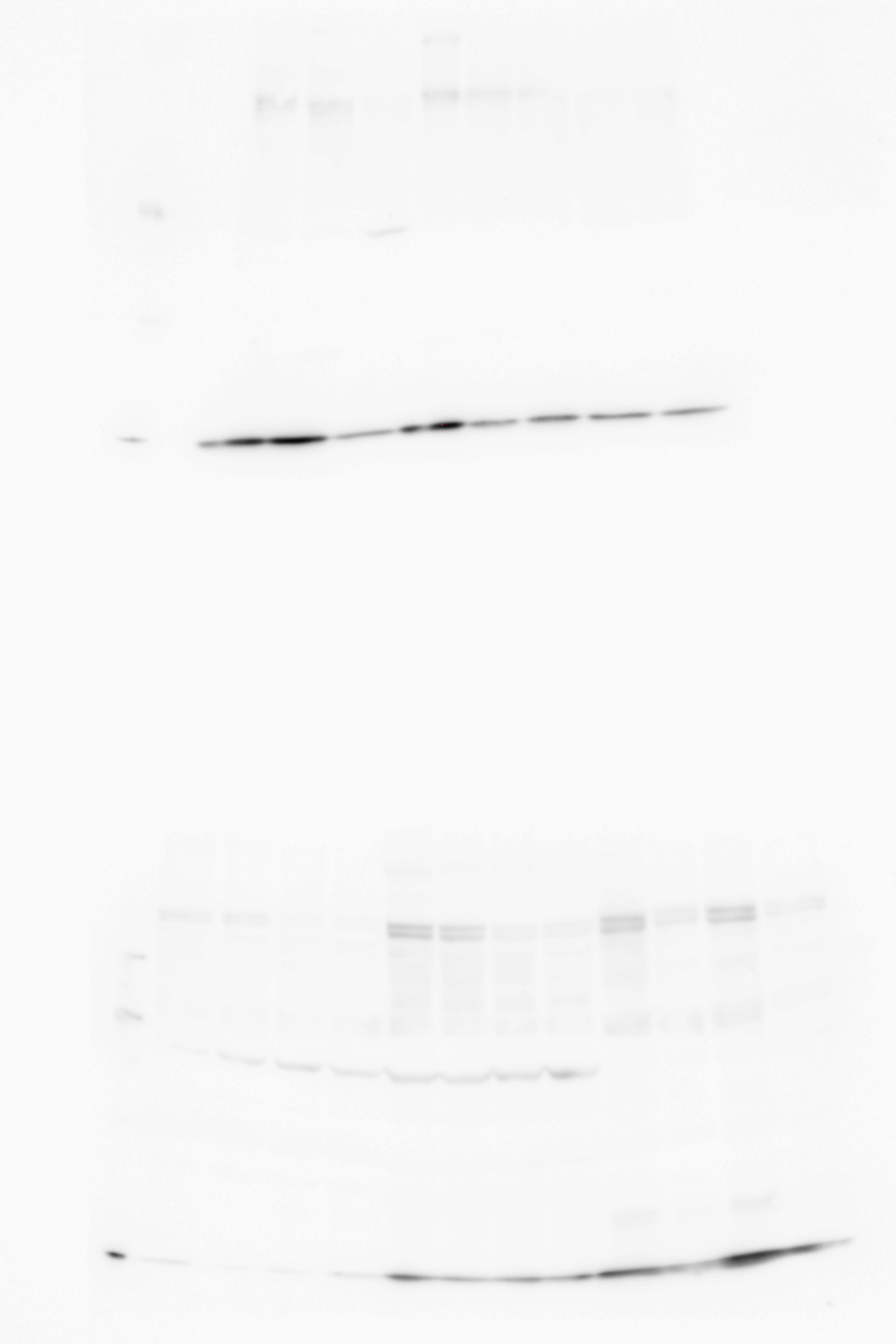

Supplement: Figure 1—figure supplement 1—source data 1. [file elife-84618-fig1-figsupp1-data1.zip › Figure 1 Suppl. 1 source data/Figure 1-figure supplement 1C-source data2.tif]

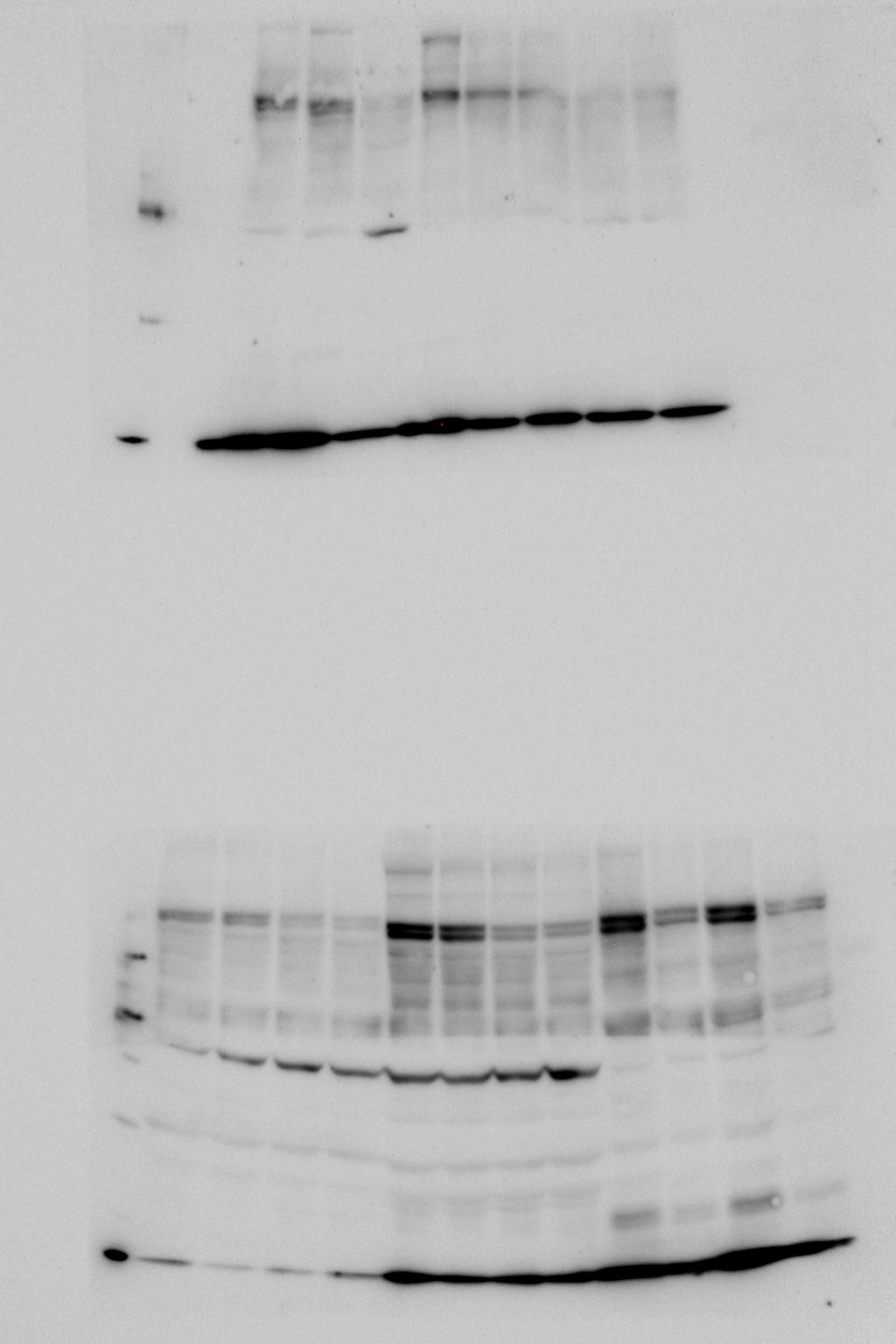

Supplement: Figure 1—figure supplement 1—source data 1. [file elife-84618-fig1-figsupp1-data1.zip › Figure 1 Suppl. 1 source data/Figure 1-figure supplement 1C-source data1.tif]

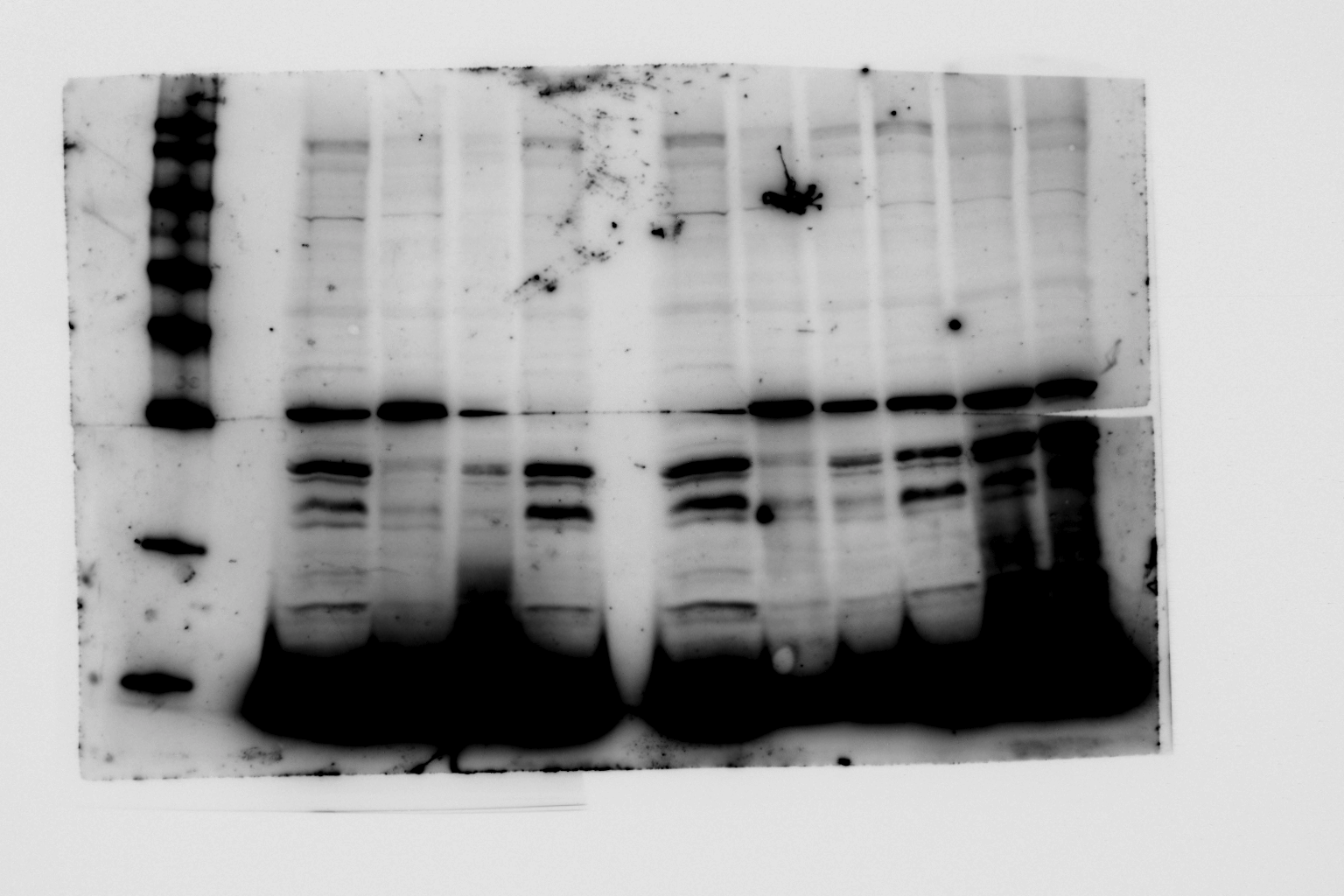

Supplement: Figure 1—figure supplement 1—source data 1. [file elife-84618-fig1-figsupp1-data1.zip › Figure 1 Suppl. 1 source data/Figure 1-figure supplement 1C-source data5.tif]

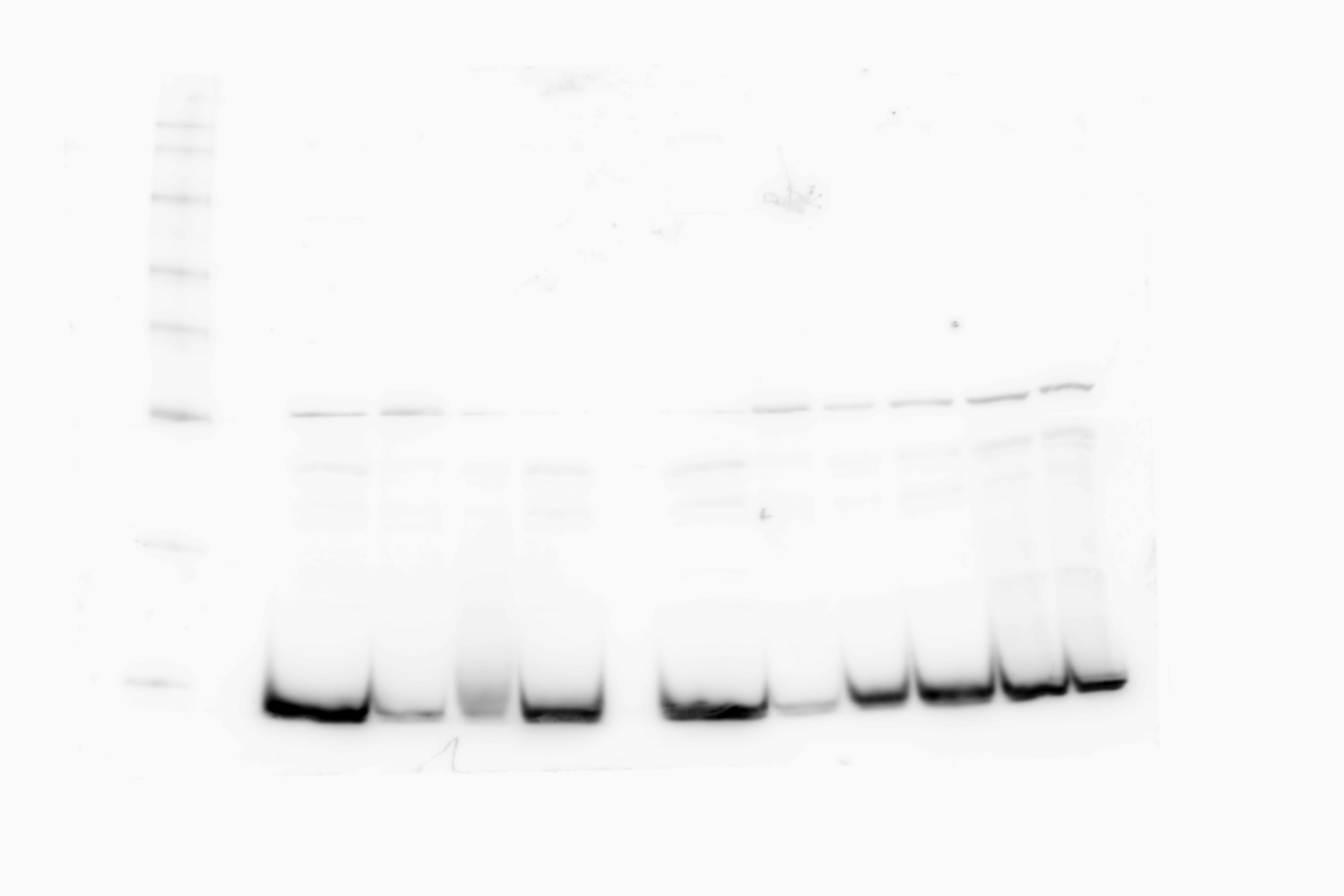

Supplement: Figure 1—figure supplement 1—source data 1. [file elife-84618-fig1-figsupp1-data1.zip › Figure 1 Suppl. 1 source data/Figure 1-figure supplement 1C-source data6.tif]

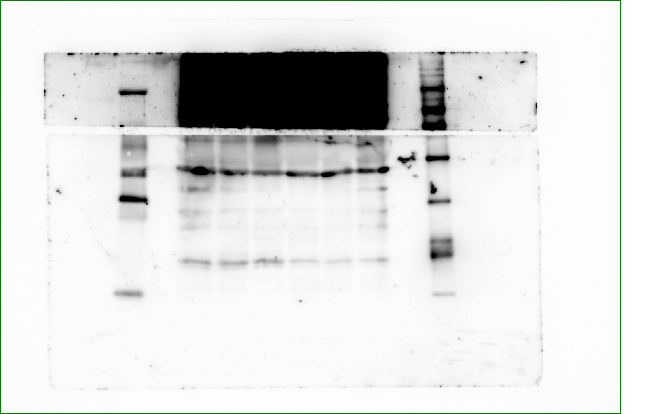

Supplement: Figure 1—figure supplement 5—source data 1. [file elife-84618-fig1-figsupp5-data1.zip › Figure 1 Suppl. 5 source data/Figure 1-figure supplement 5C-source data3.tif]

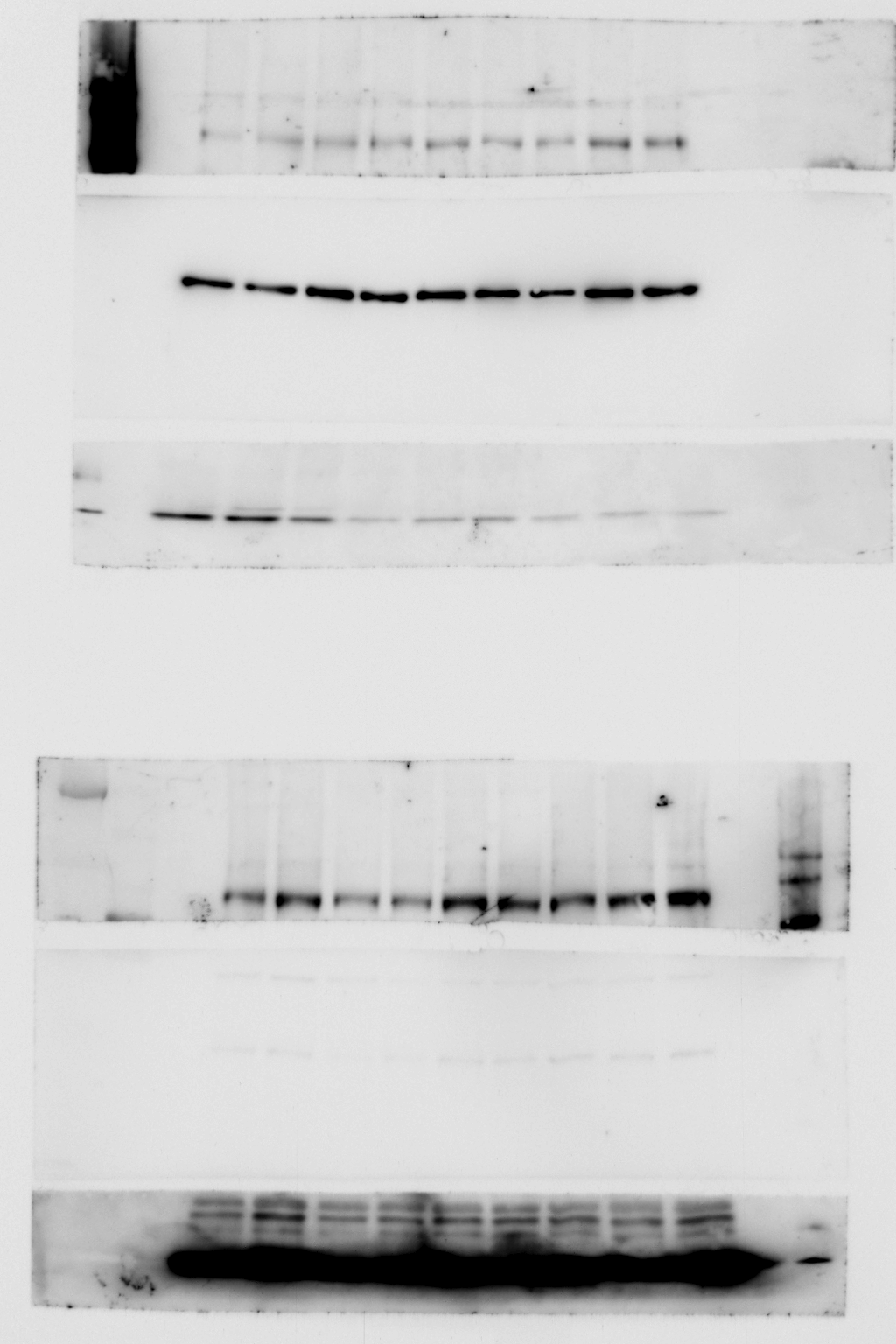

Supplement: Figure 3—figure supplement 1—source data 1. [file elife-84618-fig3-figsupp1-data1.zip › Figure 3 Suppl. 1 source data/Figure 3-figure supplement 1B-source data3.tif]

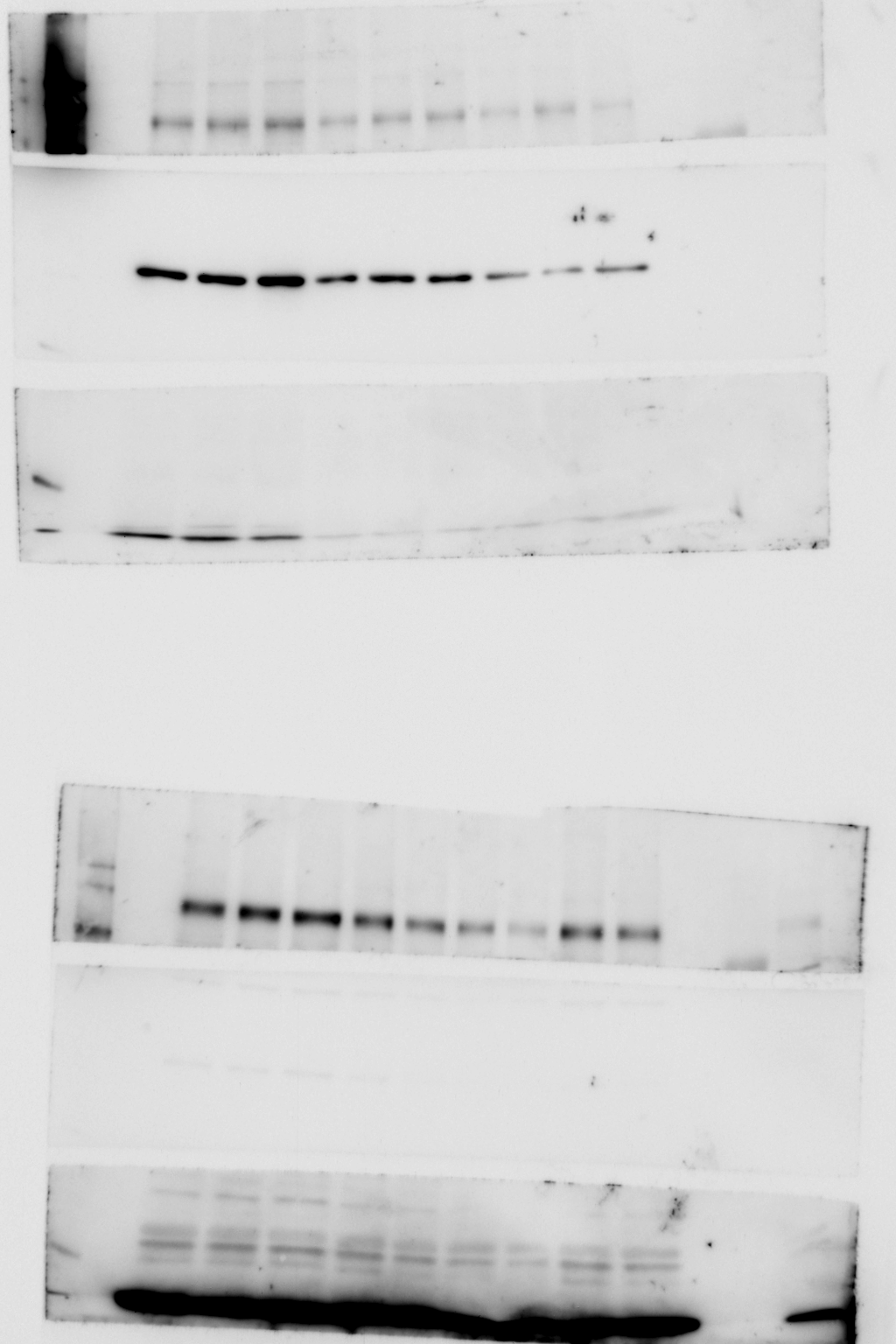

Supplement: Figure 3—figure supplement 1—source data 1. [file elife-84618-fig3-figsupp1-data1.zip › Figure 3 Suppl. 1 source data/Figure 3-figure supplement 1B-source data1.tif]

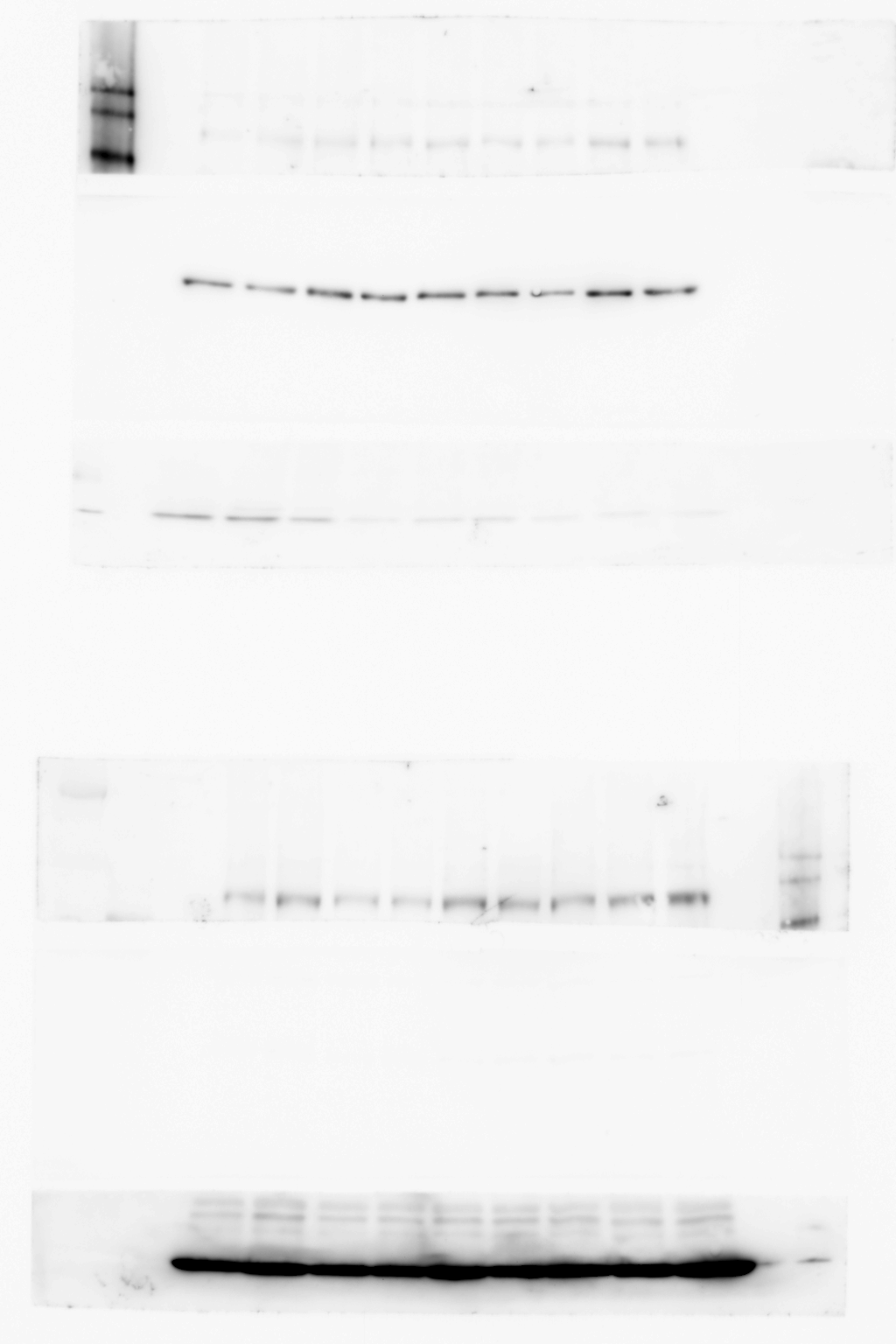

Supplement: Figure 3—figure supplement 1—source data 1. [file elife-84618-fig3-figsupp1-data1.zip › Figure 3 Suppl. 1 source data/Figure 3-figure supplement 1B-source data4.tif]

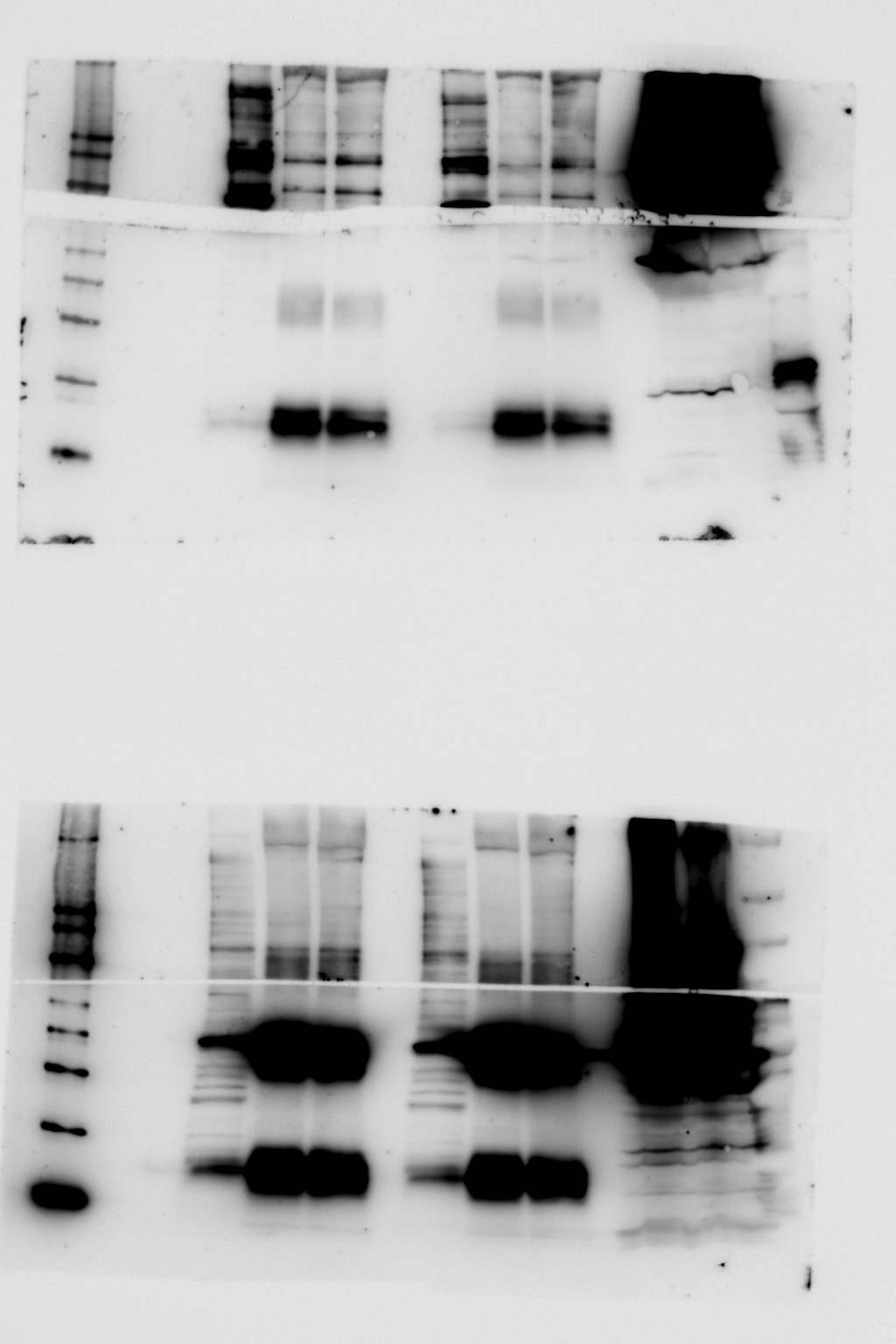

Supplement: Figure 3—figure supplement 1—source data 1. [file elife-84618-fig3-figsupp1-data1.zip › Figure 3 Suppl. 1 source data/Figure 3-figure supplement 1C-source data2.tif]

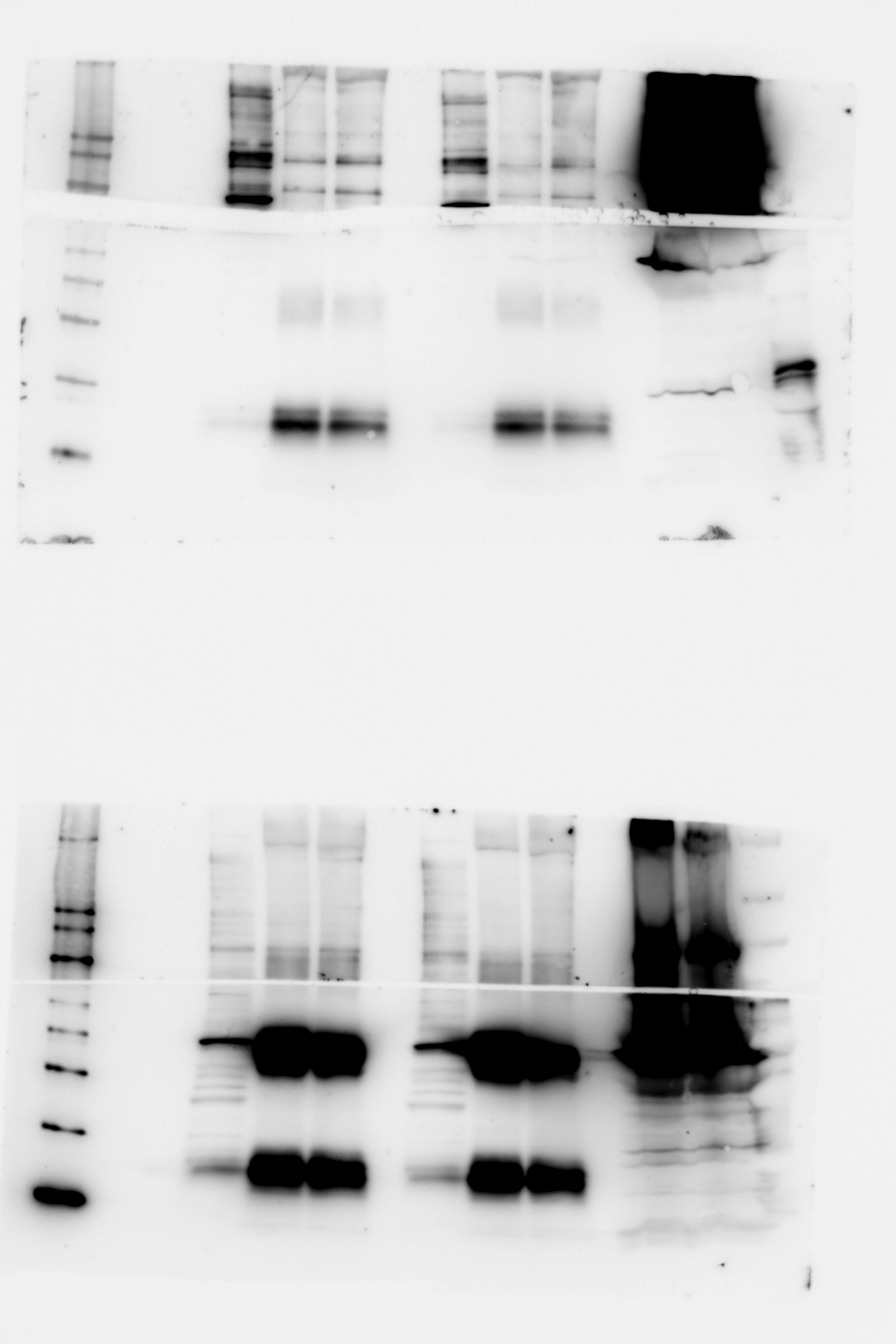

Supplement: Figure 3—figure supplement 1—source data 1. [file elife-84618-fig3-figsupp1-data1.zip › Figure 3 Suppl. 1 source data/Figure 3-figure supplement 1C-source data1.tif]

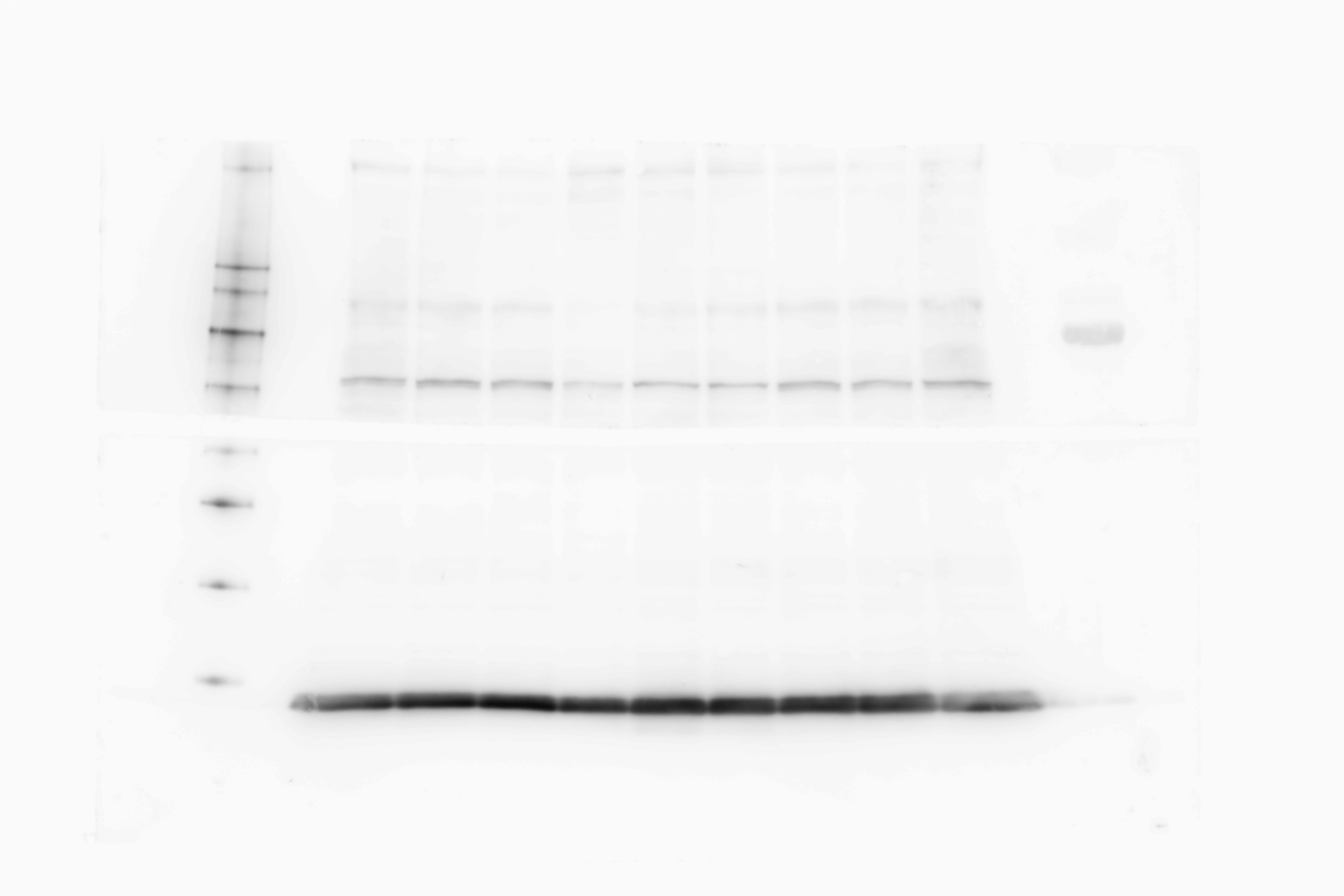

Supplement: Figure 3—figure supplement 2—source data 1. [file elife-84618-fig3-figsupp2-data1.zip › Figure 3 Suppl. 2 source data/Figure 3-figure supplement 2D-source data2.tif]

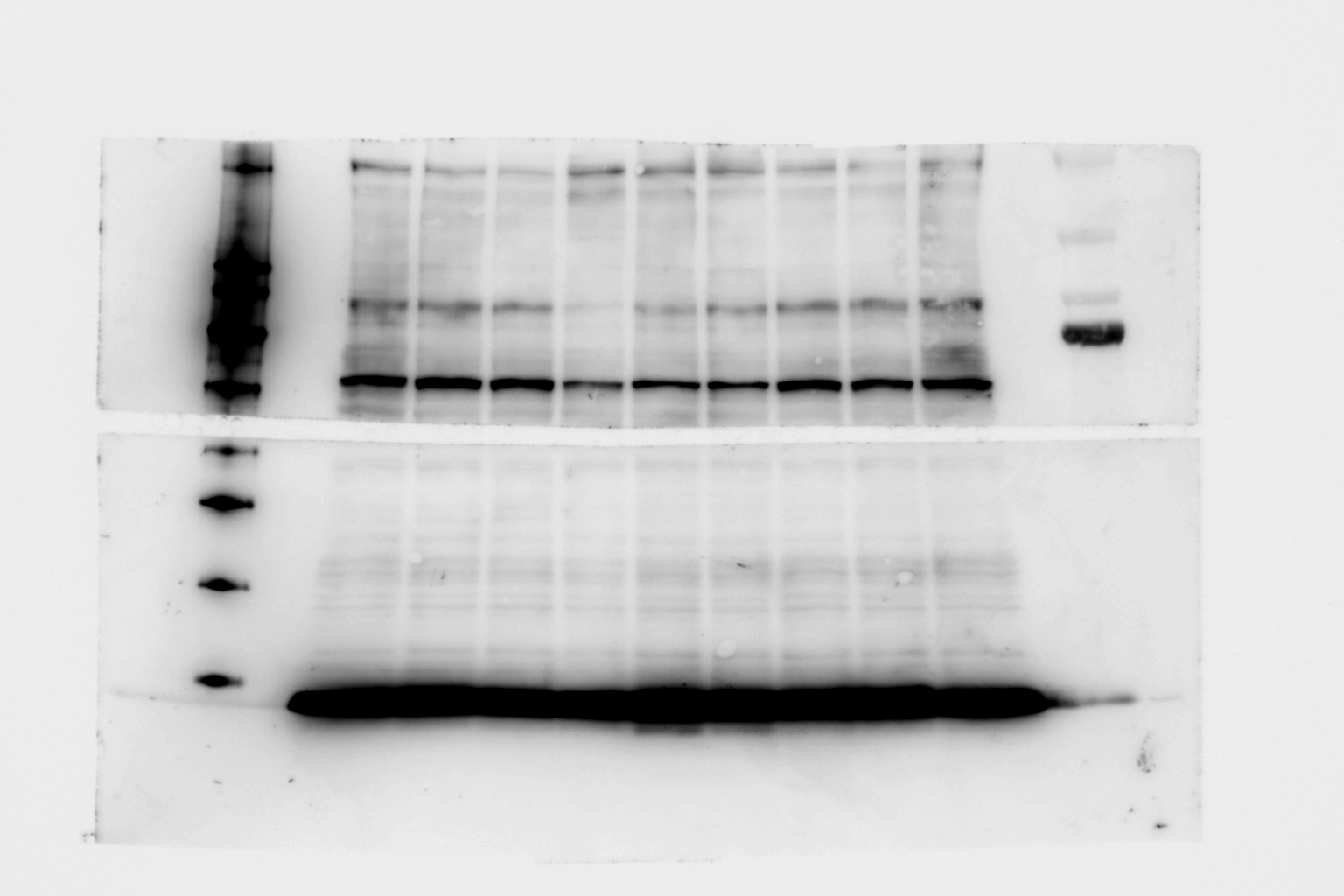

Supplement: Figure 3—figure supplement 2—source data 1. [file elife-84618-fig3-figsupp2-data1.zip › Figure 3 Suppl. 2 source data/Figure 3-figure supplement 2D-source data1.tif]

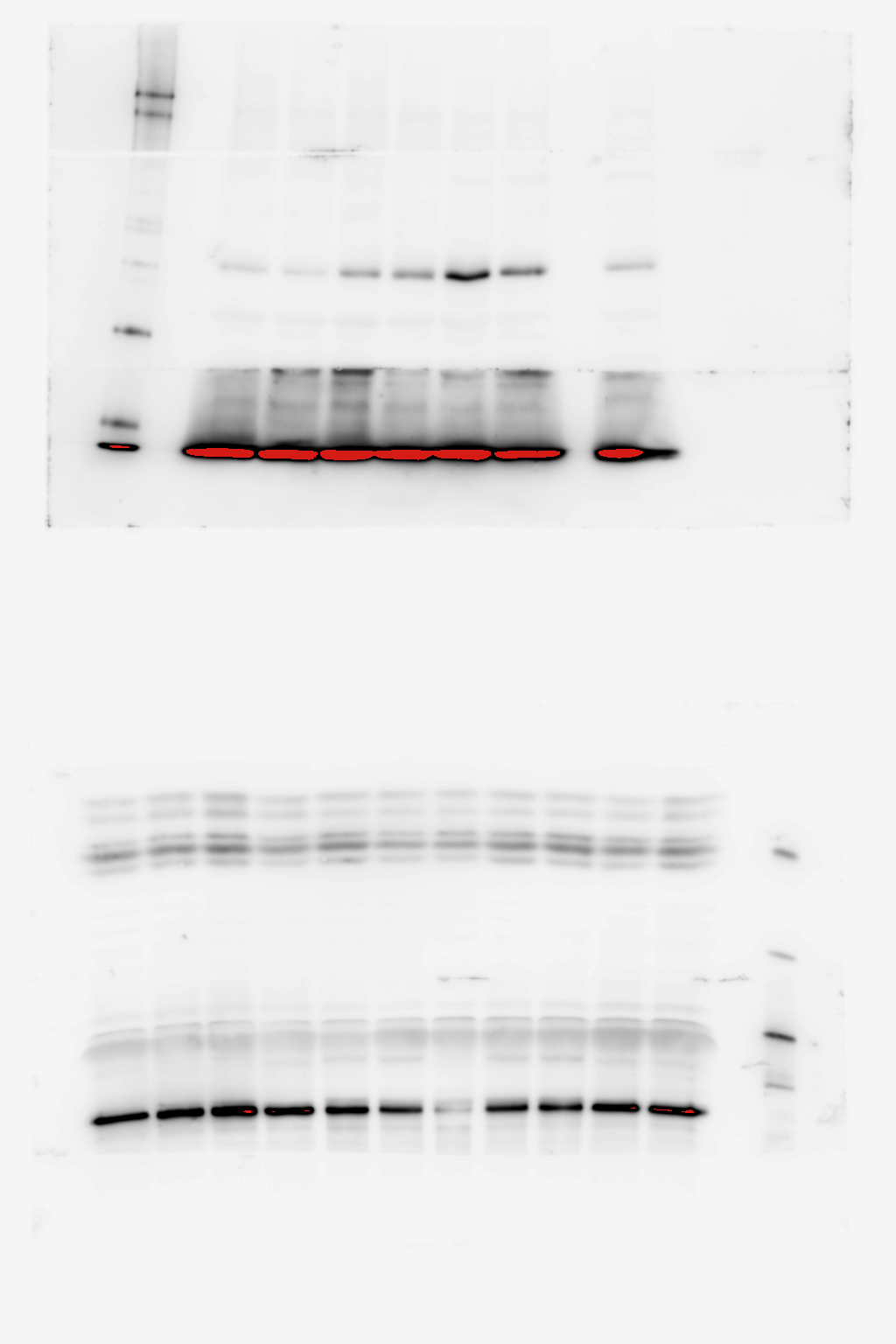

Supplement: Figure 5—source data 1. [file elife-84618-fig5-data1.zip › Figure 5 source data/Figure 5D-source data1.tif]

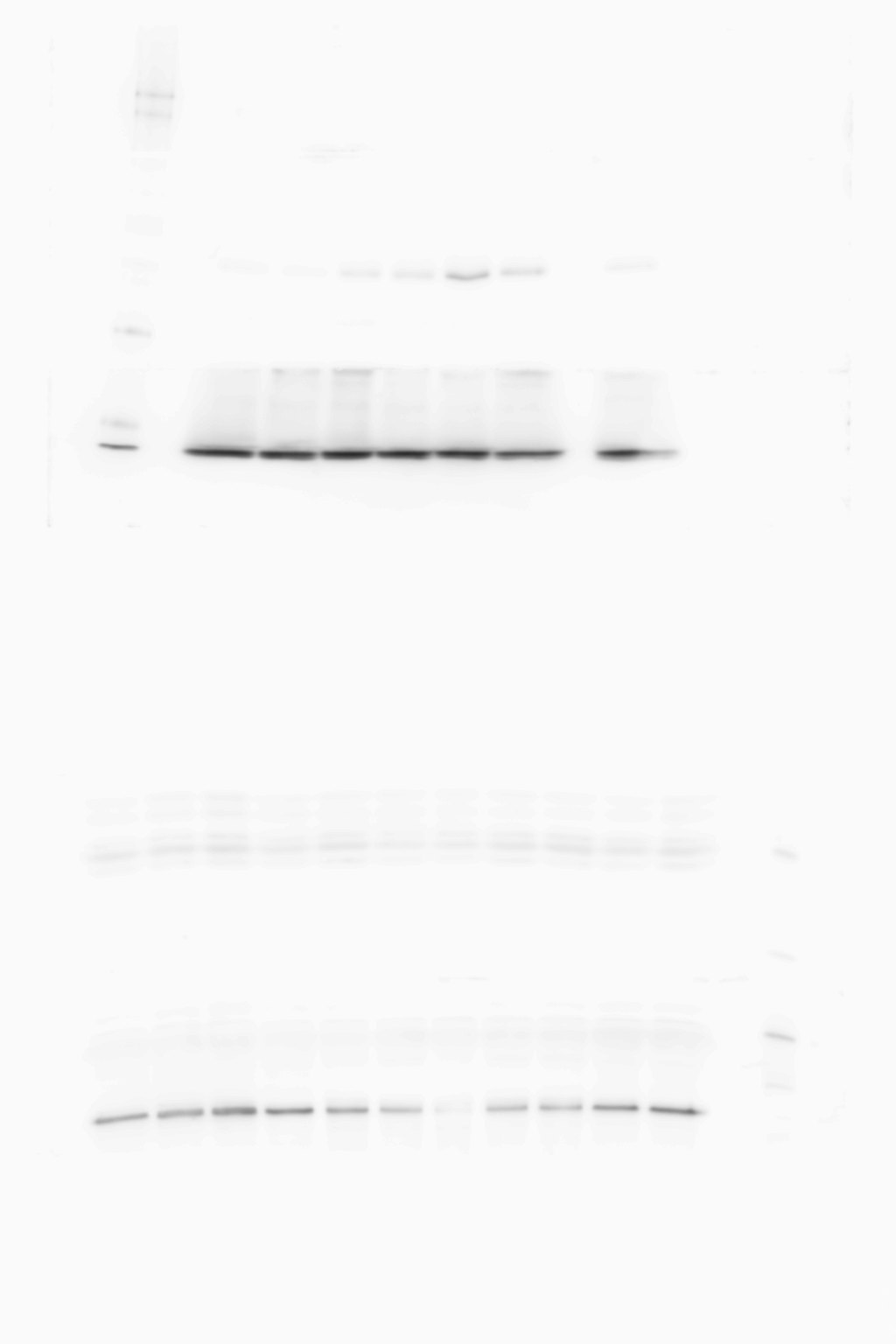

Supplement: Figure 5—source data 1. [file elife-84618-fig5-data1.zip › Figure 5 source data/Figure 5D-source data2.tif]

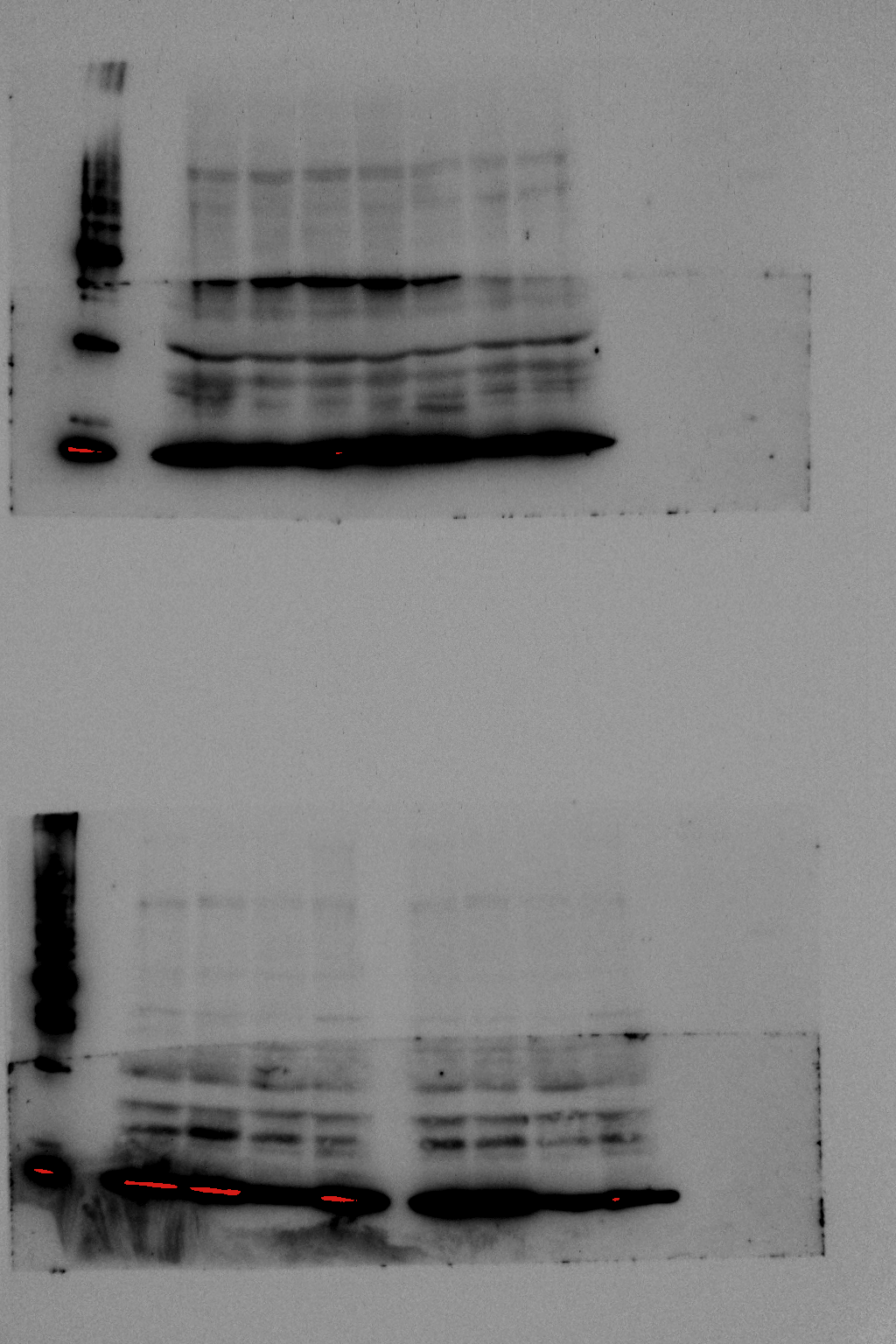

Supplement: Figure 6—source data 1. [file elife-84618-fig6-data1.zip › Figure 6 source data/Figure 6C-source data1.tif]

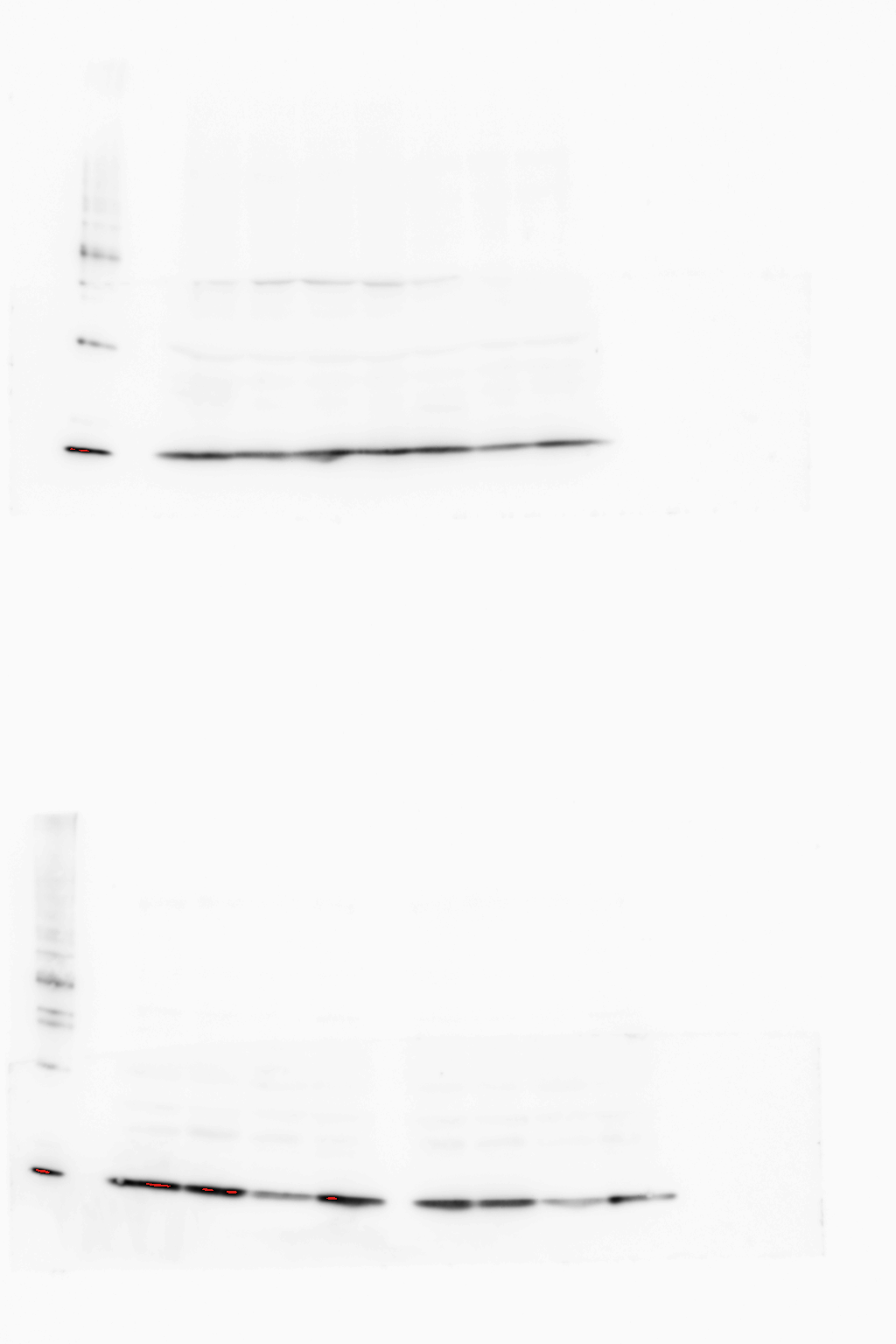

Supplement: Figure 6—source data 1. [file elife-84618-fig6-data1.zip › Figure 6 source data/Figure 6C-source data2.tif]
