# Supplementary material for: LSD1 defines the fiber type-selective responsiveness to environmental stress in skeletal muscle
Source: eLife. 2023 Jan 25;12:e84618. doi: 10.7554/eLife.84618 (PMC9876571; doi:10.7554/eLife.84618)
Supplement: Supplementary file 5. [file elife-84618-supp5.pdf]

Fig. 3E

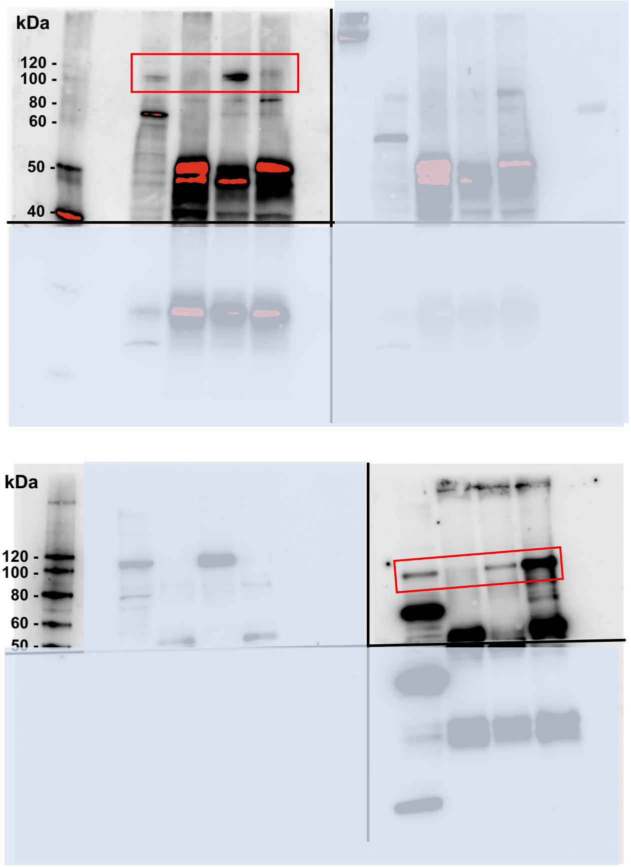

Fig. 5D

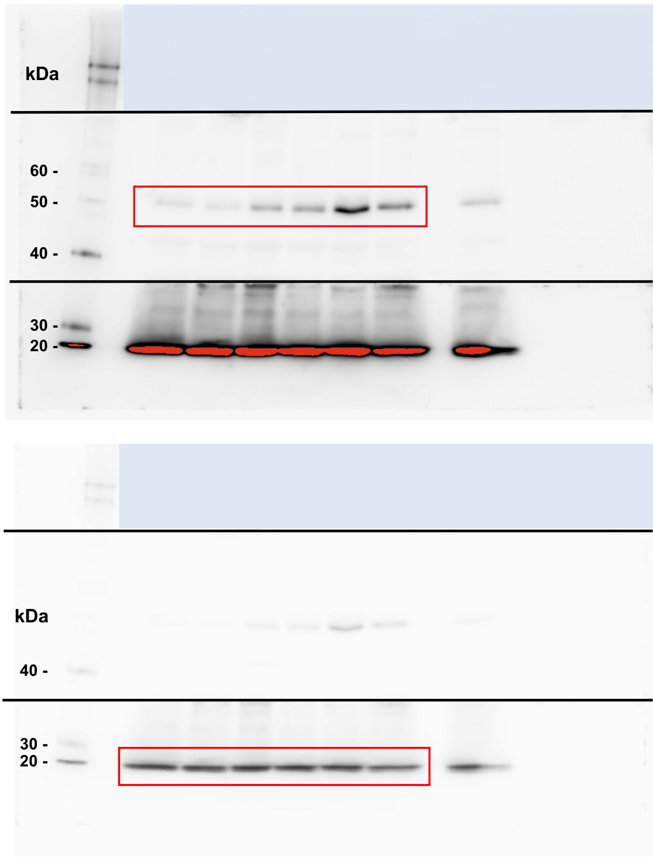

Fig. 6C

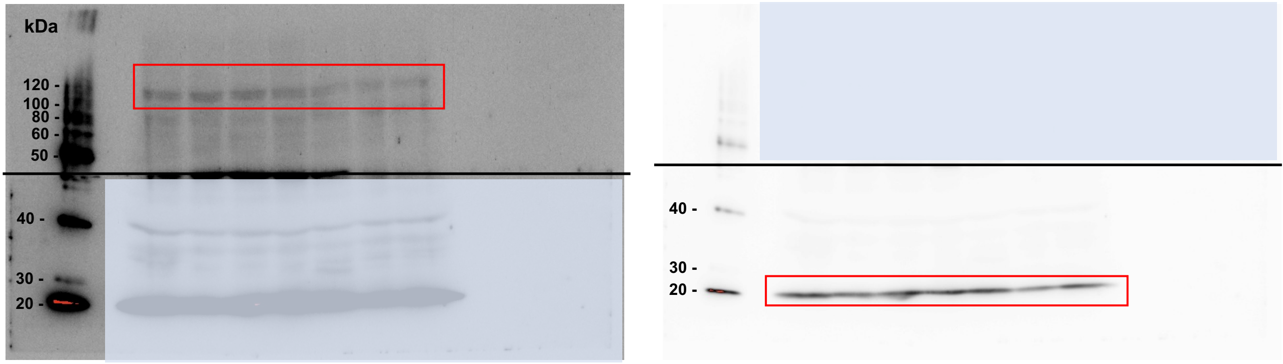

Full uncropped blots

Figure 1-figure supplement 1C

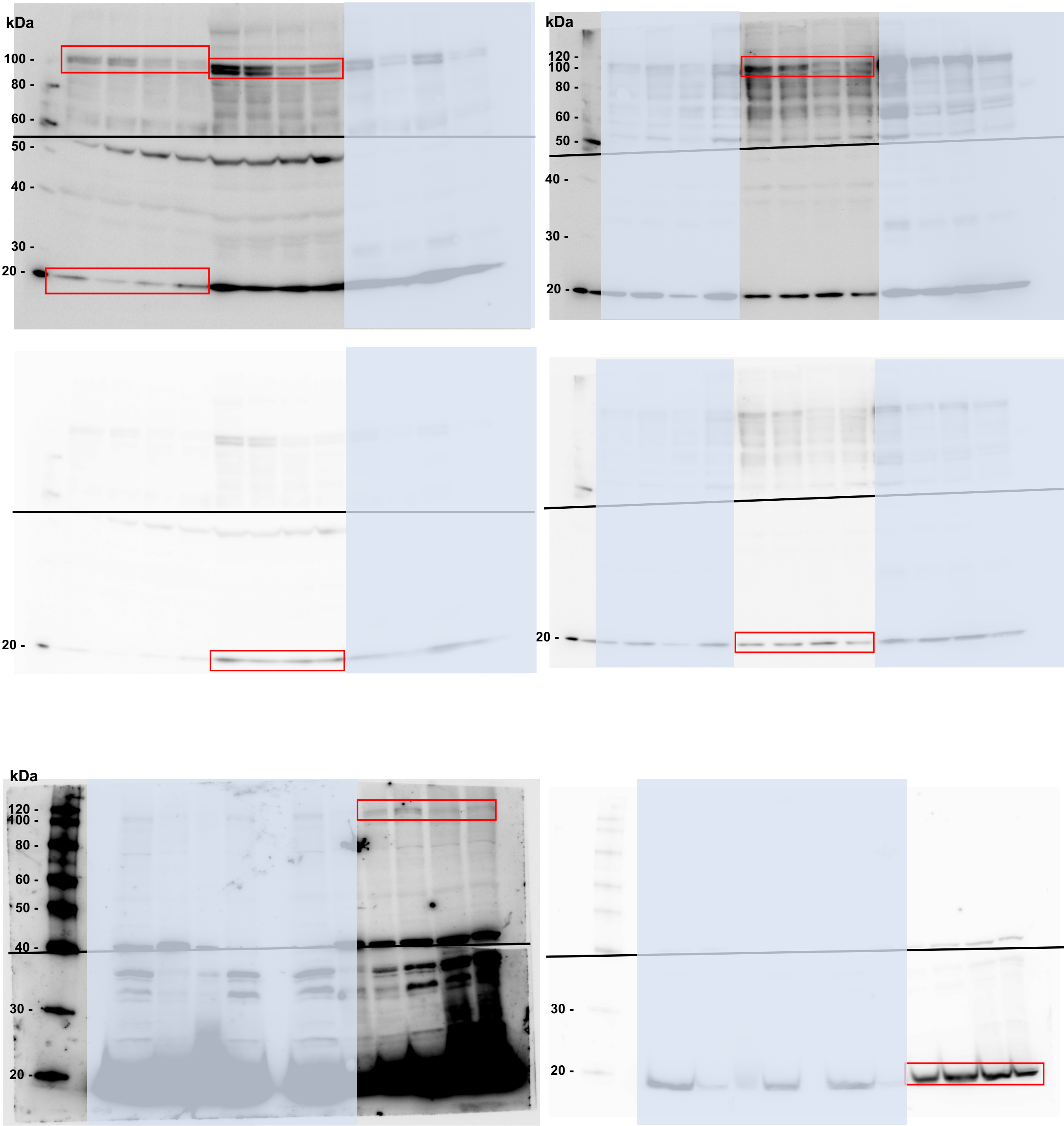

Full uncropped blots

Figure 1-figure supplement 5A

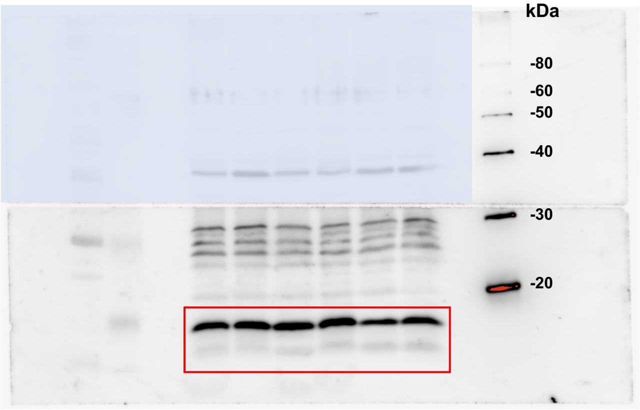

Figure 1-figure supplement 5B

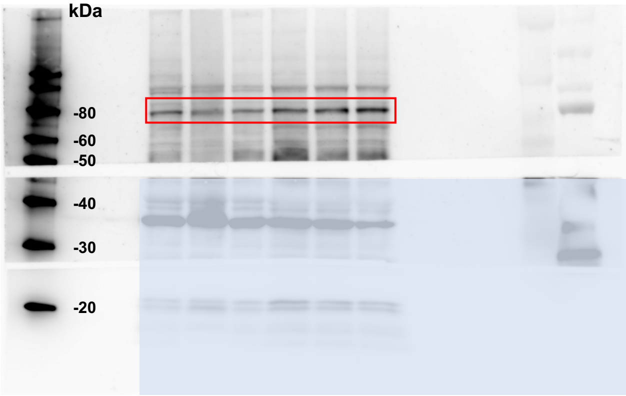

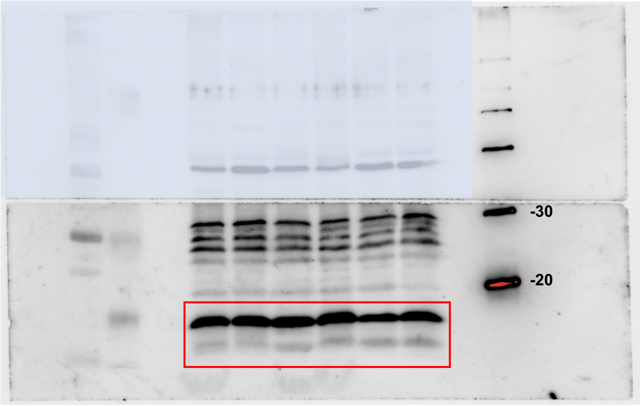

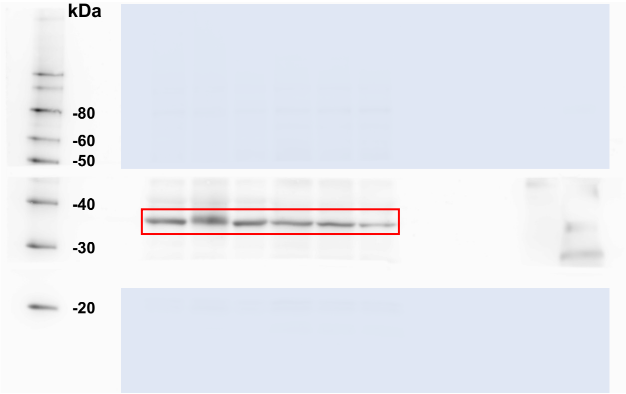

Full uncropped blots

Figure 1-figure supplement 5C

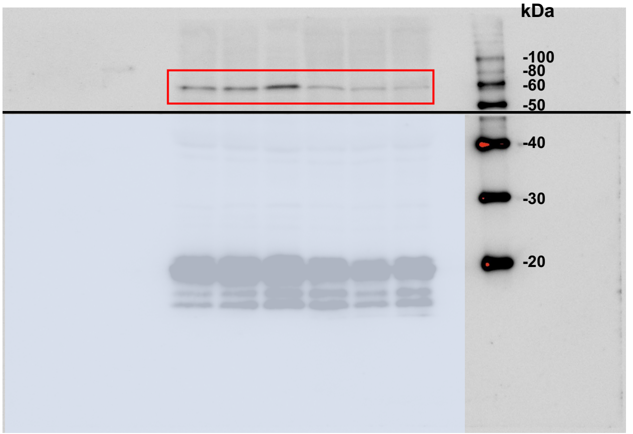

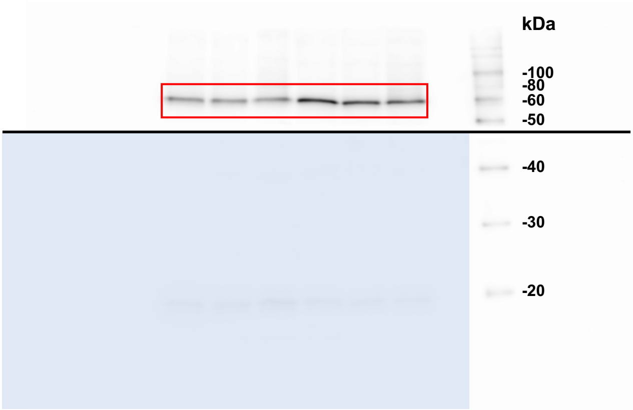

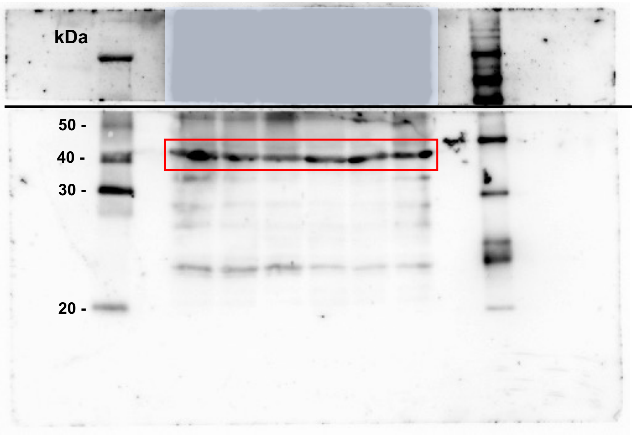

Figure 1-figure supplement 5D

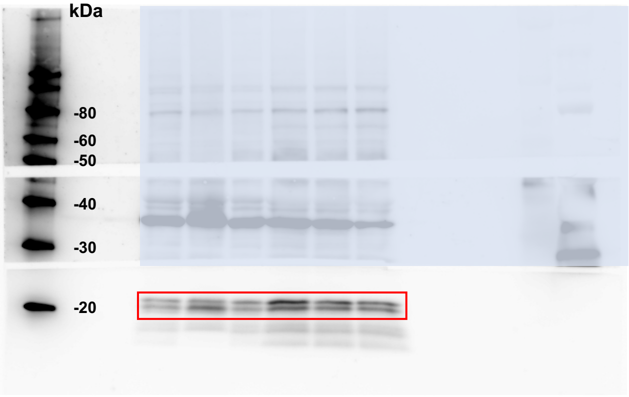

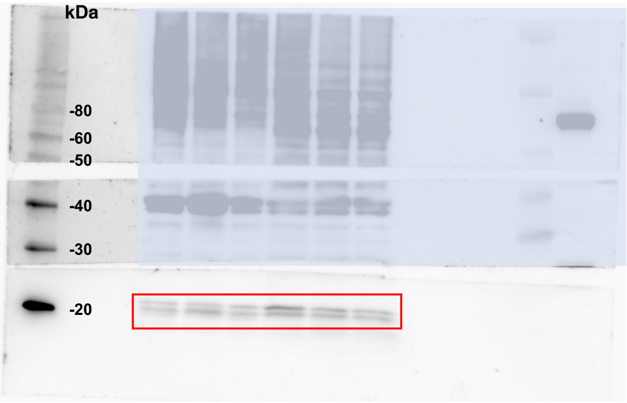

Full uncropped blots

Figure 3-figure supplement 1B

Nuclear fraction Cytoplasmic fraction

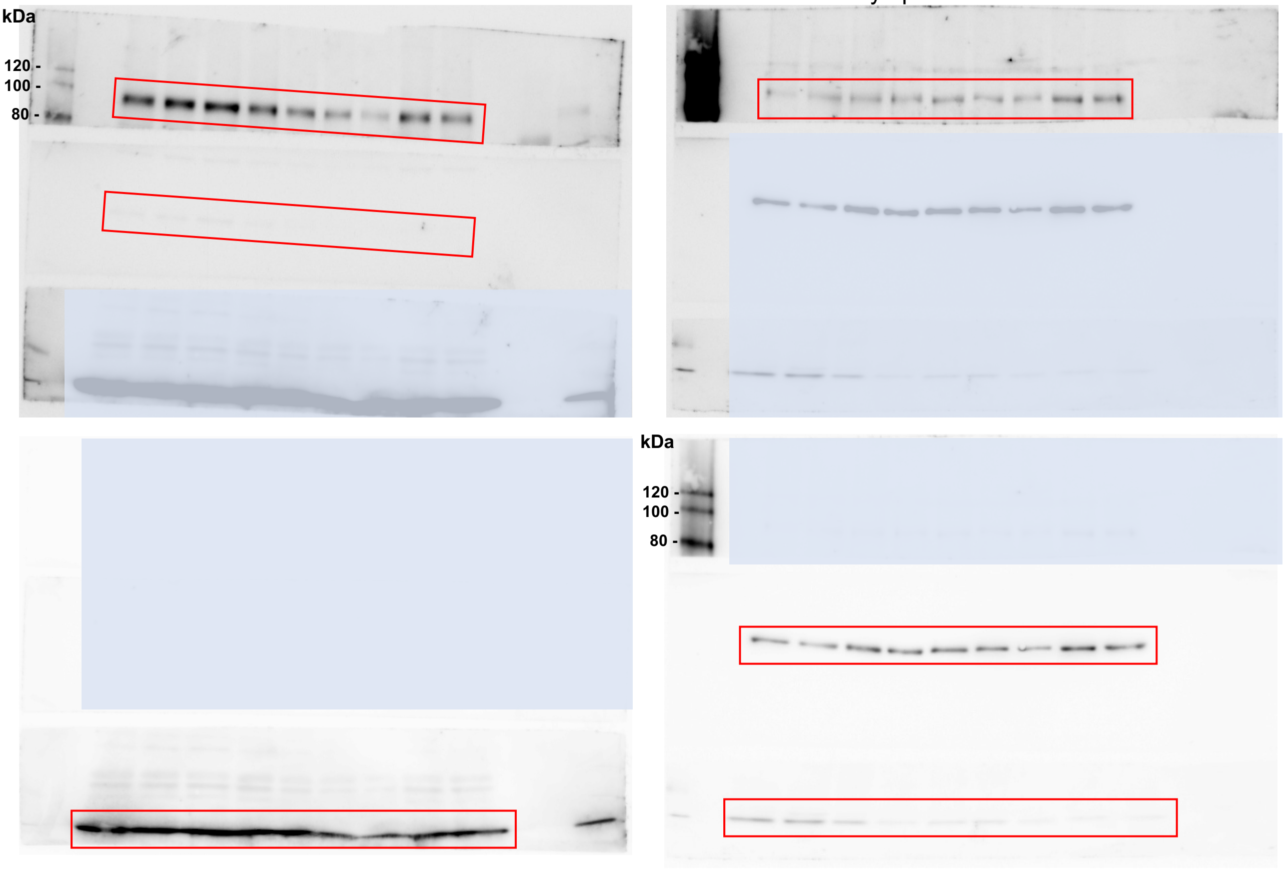

Figure 3-figure supplement 1C

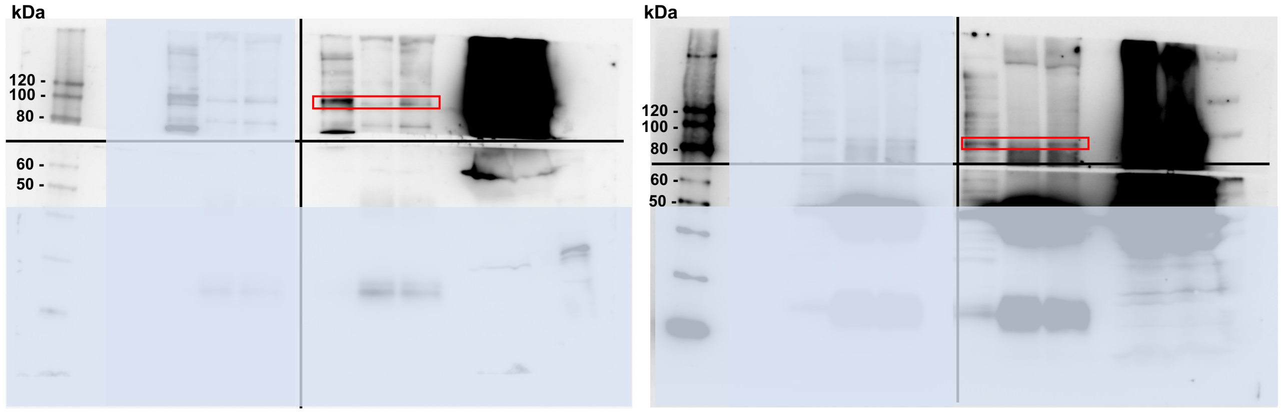

Full uncropped blots

Figure 3-figure supplement 2A

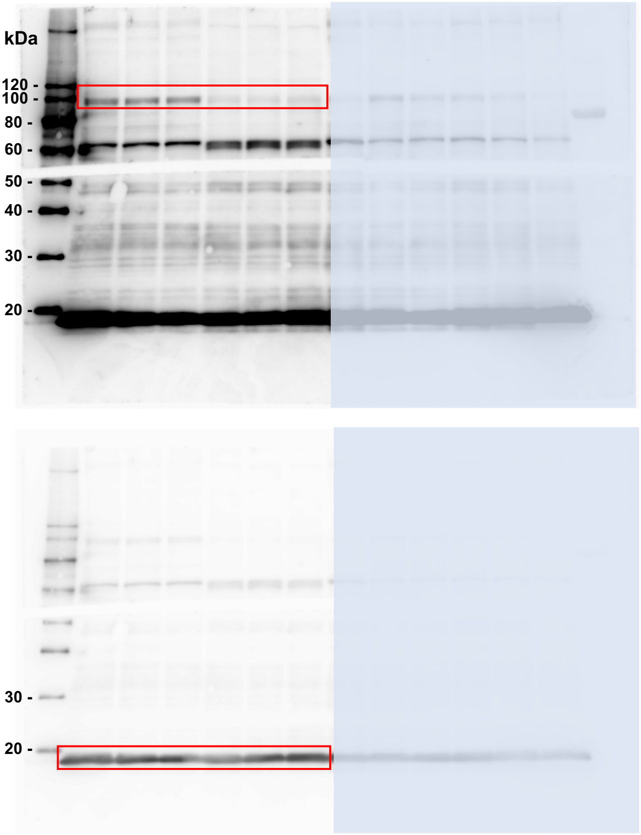

Figure 3-figure supplement 2D

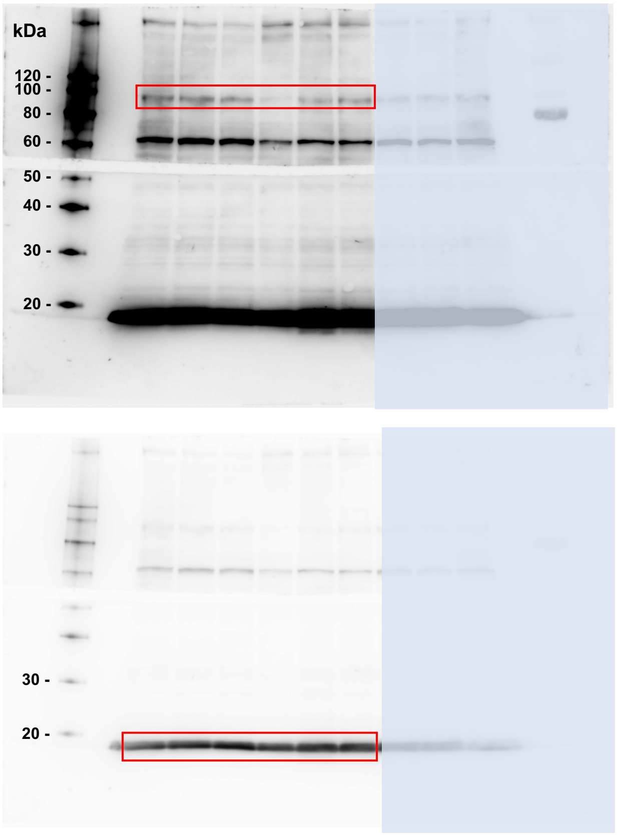

Figure 5-figure supplement 1D

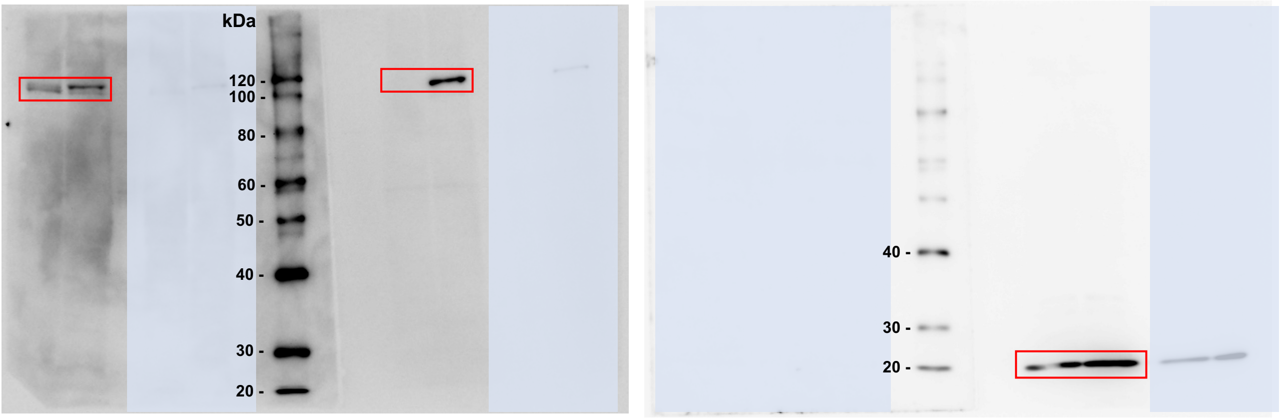

Full uncropped blots
